# The association between older adult abuse and emergency department attendance and mental health service use

**DOI:** 10.3389/fsoc.2026.1690455

**Published:** 2026-10-07

**Authors:** Sharon Sondh, Christoph Mueller, Lifang Li, Angus Roberts, Harsharon Kaur Sondh, Robert Stewart

**Affiliations:** 1 Institute of Psychiatry, Psychology and Neuroscience, King’s College London, London, United Kingdom; 2 South London and Maudsley NHS Foundation Trust, London, United Kingdom; 3 School of Journalism and Communication, Sun Yat-sen University, Guangzhou, Guangdong, China

**Keywords:** abuse, emergency department, mental health services, natural language processing, older adults

## Abstract

**Introduction:**

Older adult abuse is associated with a higher risk of mental health disorders but to date older adult abuse has not been investigated as a predictor of mental healthcare outcomes. The purpose of this study was to systematically ascertain recorded abuse and analyse its consequences in older people receiving mental healthcare.

**Methods:**

In a large South London mental healthcare data platform, recorded older adult abuse was ascertained using natural language processing algorithms during the first 3 months of contact and investigated in relation to risk of subsequent emergency department attendance and 12-month mental health service contacts. Analyses were stratified by gender, ethnicity, marital status, and deprivation.

**Results:**

Of 14,591 patients seen by mental health services, 8.4% were recorded as having experienced some form of abuse. Physical abuse was the most common type and financial abuse the least common. In adjusted models, only emotional abuse was associated with emergency department attendance (adjusted hazard ratio: 1.13, 95% CI: 1.02–1.26), but all abuse subtypes were associated with higher mental health service contact, most strongly for recorded sexual abuse (adjusted incidence rate ratio: 2.35, 95% CI: 1.93–2.86).

**Conclusion:**

Abuse experiences are commonly recorded in older adults receiving mental healthcare and are associated with a higher level of mental health service contact. With the use of natural language processing, we were able to successfully gain a deeper insight into the outcomes of abuse experienced by adults referred to a range of services.

## 1 Introduction

Older adult abuse is recognised as a global health concern and is linked to worse psychological and physical health (Gagnon et al., 2023). Studies estimate that 10% of individuals over the age of 65 encounter some form of abuse (Patel et al., 2021). Abuse is a single or repeated act and can take many forms including physical, sexual, psychological/emotional, or financial (World Health Organisation, 2024). Physical abuse has been defined as intended use of physical force which results in discomfort, injury, or damage; this type of abuse can involve kicking, slapping, hitting, burning, and pushing (Rosen et al., 2018). Psychological, emotional, and sexual abuse involves both verbal and non-verbal behaviours that are intended to cause distress (Rosen et al., 2018). Examples of these are isolation from others, harassment, intimidation, verbal berating, and/or any behaviours of a sexual nature that are unwanted and non-consensual. Financial abuse involves unlawful use of a person’s money, possessions, or properties (Rosen et al., 2018). Other examples of this kind of abuse include stealing money and coercing an older adult into editing wills or signing documents (Rosen et al., 2018). These abuse types can be carried out individually but more often co-occur and are interrelated to each other, heightening the impact on older adults (Fraga Dominguez et al., 2022).

Elder abuse may have substantial impacts on a range of both physical and mental health outcomes. A 2019 systematic review of observational research highlighted associations of elder abuse with outcomes including premature mortality, higher healthcare utilisation, and multiple physical and psychological symptoms (Yunus et al., 2019). A recent (2025) systematic review further confirmed adverse mental health outcomes, including depression, anxiety, suicidal ideation, and sleep difficulties (Fang et al., 2025). In a large national UK survey, violence in the past year had been experienced by 2% of older adults, and this was associated with a 2-fold increase in common mental disorder (Fadeeva et al., 2024). Elder abuse in a large Indian survey confirmed associations with worse mental health and wellbeing, as well as a range of physical health symptoms and higher healthcare use (Pengpid and Peltzer, 2021). A 2023 review highlighted the global importance of elder abuse, its widespread health impacts, and the importance of surveillance and policy interventions (Alias et al., 2023). A large part of the evidence has been drawn from cross-sectional data (Yunus et al., 2019), and bidirectional causal pathways may underlie some of the health associations. For example, poor self-rated health in older people was found to be a risk factor for future abuse (physical, emotional, and financial) (Burnes et al., 2021), and caregiving has also been found to be a risk factor for experiencing emotional and financial abuse (Nyarko-Odoom et al., 2023).

A 2024 synthesis of qualitative studies of experiences and perceptions confirmed reports of physical and psychological burdens in older adults experiencing abuse, as well as financial stress, and highlighted the importance of social support and abuse perceptions in both the emergence of abuse and help-seeking (Li et al., 2024). However, barriers to recognition have been reported. Specifically, a 2020 scoping review of emergency department presentations concluded that elder abuse was under-reported compared to community estimates and highlighted poor training reported by healthcare professionals and other barriers to care (Mercier et al., 2020). This under-recognition may be a driver of higher healthcare use. For example, in a study using 10 years of US Medicare data, older adults reporting abuse had higher emergency department use and hospitalisation, particularly around the time of the reported abuse (Rosen et al., 2023) with subsequent analysis confirming substantially higher healthcare costs (Zhang et al., 2024). Recent research using a large nationally representative general population sample (Fadeeva et al., 2024) has identified that abuse in older adults is associated with poor mental health. However, it remains to be clarified whether this results in higher emergency department attendances or greater use of mental health services.

Natural language processing (NLP) has transformed large-scale data availability when applied to the growing volumes of information sourced from electronic health records (EHRs) (Velupillai et al., 2018), particularly in mental healthcare where information of interest is more often in text than structured fields (Stewart and Velupillai, 2021). Through NLP, free text records can be processed at scale and valuable information extracted that would otherwise be inaccessible for analysis (Stewart et al., 2024). NLP has also been successfully deployed to ascertain diverse constructs including symptom profiles (Jackson et al., 2018), occupation (Leightley et al., 2020), medication (Chaturvedi et al., 2020), and pain (Chaturvedi et al., 2023). This is exemplified in the data used for the analyses presented here, which were made available through an NLP application designed to ascertain experienced and perpetrated violence recorded in mental healthcare EHRs. Large language models were deployed to extract keywords linked to violence and abuse so that instances could be extracted alongside relevant features [e.g., victimisation versus (vs.) perpetration; type of violence] (Li et al., 2026). An earlier version of this application had already successfully extracted data on domestic, physical, and sexual domains (Botelle et al., 2022). This study showcases the extended coverage of updates, which better triangulate different features (e.g., direction and type) as well as increasing the coverage to include psychological and financial abuse as violence types. Applying these newly available resources, our study aimed to address the earlier mentioned evidence gap and gain a better insight into the outcome of abuse faced by older adults and the consequent use of services.

## 2 Materials and methods

### 2.1 Data source

A retrospective cohort study was assembled using the Clinical Record Interactive Search (CRIS) platform to access deidentified electronic healthcare records from the South London and Maudsley NHS Foundation Trust (SLaM) (Perera et al., 2016). SLaM is one of the largest specialist mental healthcare providers in Europe, including services for older people and those with dementia. It serves a population of approximately 1.36 million residents across four South-East London boroughs (Lambeth, Lewisham, Croydon, and Southwark). CRIS was developed in 2007–2008 (Perera et al., 2016), has supported over 400 research publications to date, and has received ethics approval as a database for secondary analysis ([Oxford Research Ethics Committee C (reference 23/SC/0257) (Chaturvedi et al., 2023)]. Data are sourced both from structured fields and from free text (e.g., clinical case notes and correspondence), the latter through natural language processing (NLP) algorithms developed using General Architecture for Text Engineering (GATE) (Cunningham et al., 2013; Mueller et al., 2021) and other software. All NLP algorithms plus their performance data are listed on an open-source online catalogue (South London and Maudsley Biomedical Research Centre, 2024), and these include algorithms for ascertaining recorded abuse as described below. CRIS has also been linked to a range of other data resources, including national Hospital Episode Statistics, used in this study, as described below.

### 2.2 Study sample

All patients with an accepted referral and at least one face-to-face appointment with SLaM’s older adult mental health services between 1 January 2007 and 31 December 2022 were ascertained. The index date for exposure definition and commencement of follow-up was the 3-month point after the first face-to-face contact with older adult mental health services. We extracted the following patient characteristics at the index date: age, gender, ethnicity (dichotomised into White and non-White), marital status (dichotomised into married/cohabiting and non-cohabiting), and neighbourhood socioeconomic status measured by the Index of Multiple Deprivation (IMD)(Noble et al., 2007), which is a Census-derived measure available at the level of the Lower Super Output Area (standard administrative units with approximately 1,500 residents). For stratification purposes, we divided the cohort into IMD tertiles.

### 2.3 Exposure: abuse/violence

As described above, an NLP application to identify and classify different types of abuse had been developed. Building on earlier NLP work that identified experiences of violence/abuse in mental health records (Botelle et al., 2022) this updated version, which has been described in detail (Li et al., 2026), broadened detected violence from the original focus on physical and sexual abuse to also include psychological/emotional and financial abuse. To achieve this, 6,500 text instances were extracted from the CRIS SLaM system of which 90% were used for training and 10% for blind testing. Instances were defined from a gazetteer of violence-related terms and were extracted from free text within patient medical records and annotated using a guideline by two research assistants. The annotation guidelines contained clear examples and definitions and were updated regularly. Weekly meetings with the wider research group (which involved domain experts) were conducted to ensure annotations were done accurately and any misunderstandings were discussed. These instances were then used to fine-tune a multi-label BERT model which performed strongly in categories with sufficient training data. We selected a fine-tuned BERT model as a previous study had shown that it performs more accurately in comparison with the more traditional symptom-detection approaches (including support vector machines) (Mascio et al., 2020). In particular, BioBERT offers advantages for deployment and implementation in clinical environments as the model is easy to follow and interpret as well as accessible on GitHub (Lee et al., 2020). The pre-trained BERT model was further trained using 95% of the annotated datasets, and the remaining was used for blind testing (which were balanced on gender, ethnicity, and age). The performance of the model differed in accordance with the abuse type; with the blind test capturing emotional, financial, physical, and sexual abuse very well (precision/recall estimates for 651 instances: 83%/95%, 81%/96%, 78%/91%, and 89%/55%, respectively). This specific application describes abuse types, and values applied here were emotional, financial, physical, and sexual abuse. In further independent evaluation (against manual evaluation), the precision (positive predictive value) for the presence of abuse in a random sample of 50 clinical documents ranged between 76% for physical abuse and 100% for emotional abuse.

Both violence and abuse were included as terms for NLP development. For the purpose of this manuscript, we are using the term abuse. Patients were defined with recorded abuse if this was ascertained by the NLP algorithm prior to the index date—i.e., from any time to before and up to 3 months after first face-to-face contact with older adult mental health services.

### 2.4 Outcomes: emergency department attendance and face-to-face contacts with mental health services

From linked Hospital Episode Statistics (NHS Digital, n.d), we ascertained emergency department attendances after the index date. The HES database records all emergency department attendances in all National Health Service (NHS) hospitals in England and allows tracking of patients between different hospitals and across different years. The second outcome examined was the number of face-to-face contacts with mental health services extracted via CRIS and ascertained within 12 months after index date. This analysis took into account the days individual patients were under the care of mental health services in those 12 months.

### 2.5 Statistical analyses

All statistical analyses were carried out using STATA 18 software (StataCorp, 2023). Initially, descriptive statistics were generated to compare those with/without recorded abuse. Next, Cox regression models were applied to examine the relationship between recorded abuse and emergency department attendance, in which patients were followed from the index date to their first emergency department attendance, date of death, or a censoring date on 31 December 2022. Negative binomial regression models assessed the relationship of recorded abuse with the number of mental health service face-to-face contacts. Multivariate models for both these outcomes adjusted for age, gender, ethnicity, marital status, and neighbourhood deprivation. Exposures evaluated included both any abuse and specific categories of abuse, comparing each to a control group with no recorded abuse of any type. Furthermore, we stratified the sample by gender, ethnicity, marital status, and deprivation to examine whether there were differences in outcomes in relation to recorded abuse of any type, testing this with interaction terms in the multivariate models.

## 3 Results

### 3.1 Cohort characteristics

We ascertained 14,591 patients seen by older adult mental health services in the observation period. The mean (SD) age of the cohort was 78.8 (7.5) years, and 61.5% were female. Of the 14,591 patients, 1,227 (8.4%) were recorded as experiencing abuse. In those with any recorded abuse (*n* = 1,227), physical abuse was most common (64.6%, *n* = 793), followed by emotional (47.3%, *n* = 580), sexual (12.0%, *n* = 147), and financial (7.9%, *n* = 97) abuse; 26.1% were recorded as subject to more than one form of abuse. The characteristics of the full cohort and those with/without recorded abuse are described in Table 1. Those with recorded abuse were younger, more likely to be female and from more deprived areas, and were less likely to be married or cohabiting.

**Table 1:** Characteristics of the full cohort and by whether patients were subject to abuse.

Characteristic	Full cohort (*n* = 14,591)	Any abuse experience (*n* = 1,227)	No recorded abuse (*n* = 13,364)	*p*-value
Age (mean, SD)	78.8 (7.5)	76.4 (7.6)	79.0 (7.4)	<0.001
Female gender (%)	61.5	64.4	61.1	0.003
Non-White ethnicity (%)	28.9	29.9	28.8	0.437
Married/cohabiting (%)	37.1	30.9	37.6	<0.001
Index of multiple deprivation (mean, SD)	26.7 (11.0)	29.1 (10.7)	26.5 (11.1)	<0.001
Deprivation by tertiles (%)				<0.001
Most deprived	33.3	41.6	32.6	
Middle	33.3	33.1	33.4	
Least deprived	33.3	25.3	34.1	
Types of abuse (%)				
Emotional	4.0	47.3		
Physical	5.4	64.6		
Financial	0.6	7.9		
Sexual	1.0	12.0		
More than one type of abuse	2.2	26.1		

### 3.2 Outcome associations

The median survival time to first emergency department attendance in the whole cohort was 19.4 months (interquartile range (IQR): 8.6–43.8). Time until first emergency department attendance did not differ between those experiencing abuse (19.7, 8.6–47.6 months) and those not experiencing abuse (19.4, 8.7–43.7 months; *p* = 0.284). The mean (SD) number of mental health contacts in the year after index date was 5.9 (12.2) in the full cohort, and this was higher in those with compared to without recorded abuse (mean (SD) 11.1 (17.3) and 5.4 (11.5), respectively, *p* < 0.001).

In regression models (Table 2), experience of any abuse was not significantly associated with emergency department attendance in either unadjusted or adjusted Cox regression models; however, it was significantly associated with increased mental health service contact in both unadjusted and adjusted negative binomial regression models. The absence of association with emergency department attendance was consistent in all strata. For mental health service contacts, associations with recorded abuse remained statistically significant in all strata and were significantly stronger in patients from non-White compared to White ethnic groups, in patients who were cohabiting, and in patients from least (compared to most) deprived neighbourhoods.

**Table 2:** Cox and negative binomial regression analyses of emergency department attendance and mental health service face-to-face contacts, respectively, comparing those with/without recorded abuse and stratified by gender, ethnicity, marital status, and deprivation.

Cohort/subgroup	Emergency department attendance (HR, 95% CI)			Mental health face-to-face contacts (IRR, 95% CI)		
	Unadjusted	Adjusted	*P*-value for interaction (adjusted)	Unadjusted	Adjusted	*P*-value for interaction (adjusted)
Full cohort	0.96 (0.90–1.03)	1.06 (0.99–1.14)		**1.84(1.72–1.97)**	**1.69(1.57–1.82)**	
Gender			0.516			**0.888**
Female	0.95 (0.87–1.03)	1.04 (0.95–1.14)		**1.82(1.67–1.97)**	**1.71(1.56–1.87)**	
Male	1.00 (0.89–1.12)	1.09 (0.96–1.23)		**1.87(1.67–2.10)**	**1.70(1.50–1.93)**	
Ethnicity			0.265			0.002
Non-White	1.09 (0.96–1.24)	1.13 (0.99–1.30)		**2.20(1.94–2.50)**	**2.02(1.77–2.30)**	
White	0.93 (0.86–1.01)	1.03 (0.95–1.13)		**1.70(1.57–1.85)**	**1.58(1.45–1.72)**	
Marital status			0.856			0.026
Married/cohabiting	0.98 (0.86–1.11)	1.05 (0.92–1.20)		**1.99(1.76–2.26)**	**1.93(1.70–2.20)**	
Non-cohabiting	0.98 (0.90-1.07)	1.06 (0.97–1.16)		**1.69(1.54–1.84)**	**1.61(1.47–1.76)**	
Deprivation tertiles						
Most deprived	0.95 (0.85–1.05)	1.05 (0.94–1.17)	0.930^1^	**1.61(1.45–1.79)**	**1.52(1.36–1.71)**	**0.017** ^1^
Middle	1.01 (0.89–1.14)	1.09 (0.95–1.24)	0.623^2^	**1.91(1.70–2.15)**	**1.80(1.59–2.04)**	0.483^2^
Least deprived	0.93 (0.81–1.06)	1.05 (0.90–1.22)		**2.07(1.81–2.36)**	**1.92(1.66–2.22)**	

HR: hazard ratio, IRR: incidence rate ratio. Bold font = *p* < 0.05. Adjusted models are adjusted for age, gender, ethnicity, marital status, and deprivation—unless the analysis is stratified for those variables. Interactions 1—least deprived compared to most deprived x abuse present interaction; 2—least deprived compared to moderately deprived x abuse present interaction.

In the same regression models, considering the type of recorded abuse (Table 3), an association was found between emotional abuse and increased hazard of emergency department attendance in the adjusted model but there were no other associations with that outcome. All types of abuse were significantly associated with higher mental health contacts in both models, with strongest associations seen for sexual abuse.

**Table 3:** Cox and negative binomial regression analyses of emergency department attendance and mental health service face-to-face contacts, respectively, comparing those with different types of abuse to those not experiencing abuse (*n* = 13,364).

Types of abuse compared to no abuse	A&E attendance (HR, 95% CI)		Face-to-face contacts (IRR, 95% CI)	
	Unadjusted	Adjusted	Unadjusted	Adjusted
Emotional abuse (*n* = 580)	0.95 (0.86–1.05)	**1.13(1.02–1.26)**	**2.01(1.82–2.21)**	**1.89(1.70–2.10)**
Financial abuse (*n* = 97)	1.17 (0.94–1.46)	1.04 (0.81–1.33)	**1.73(1.37–2.19)**	**1.71(1.34–2.19)**
Physical abuse (*n* = 793)	0.96 (0.88–1.04)	1.05 (0.96–1.15)	**1.86(1.71–2.02)**	**1.75(1.60–1.92)**
Sexual abuse (*n* = 147)	0.85 (0.70–1.04)	0.97 (0.79–1.20)	**2.40(2.00–2.88)**	**2.35(1.93–2.86)**
More than one type of abuse (*n* = 320)	0.95 (0.83–1.08)	1.14 (0.99–1.31)	**2.16(1.90–2.46)**	**2.14(1.86–2.45)**

HR: hazard ratio, IRR: incidence rate ratio. Bold font = *p* < 0.05. Adjusted models are adjusted for age, gender, ethnicity, marital status, and deprivation.

## 4 Discussion

In a large sample of 14,591 older people referred to mental health services, we found that approximately one in 12 (8.4%) had at least one form of experienced abuse recorded: most commonly physical abuse, followed by emotional, sexual, and financial abuse, with an important proportion (*n* = 320, 2.2%) having more than one domain of abuse recorded. While we did not find that abuse was associated with subsequent emergency department attendance (apart from emotional abuse), those experiencing abuse received more intense mental health service input. This particularly applied to those experiencing sexual abuse and, in stratified analyses, those from ethnic minority backgrounds, those married/cohabiting, and those in least deprived areas. A determining factor for this could be that there were obstacles and/or missed opportunities to disclose violence because of organisational issues such as a lack of time, discomfort, or unfamiliarity specifically in domestic violence ascertainment (Tarzia et al., 2021). Fear of disclosure could result in patients who had experienced abuse being classified in the control group, potentially attenuating the observed association.

EHRs provide a rich overview of a patient’s health journey, including possible instances of abuse, and provide novel opportunities for generating new evidence from datasets that are both large in sample size and highly detailed. When enhanced by techniques such as NLP, these naturalistic data resources can be used to highlight previously unrecognised features, allowing healthcare providers to consider details in their assessments and encourage better detection. A previous US study which used data from EHRs found associations of abuse with conditions that lead to physical vulnerability and disability (Mouton et al., 2019). That study investigated Medicare claims data from 2012 to 2014 and extracted records for 10,181 individuals. The cohort highlighted that elder abuse was associated with higher mental health needs, a similar finding to our study. We believe that our study is one of the first in the UK to ascertain abuse experiences from routine healthcare data in older patients and that our findings highlight the need for better service provision, as individuals with past experiences of abuse are often found to face challenges when using services (Bach et al., 2021). Previous research has found that older adults who have experienced abuse are more likely to receive care in the emergency department (Rosen et al., 2023); however, our study did not find such an association in most analyses. This might be due to feelings of fear, embarrassment, or guilt associated with revealing abuse or maltreatment (Wyman and Malloy, 2024). A review conducted by Cooper, Selwood et al. concluded that almost a quarter of older adults who were dependent on carers reported substantial psychological abuse and an additional fifth reported neglect; furthermore, the range of reviewed studies indicated that vulnerable older adults are at high risk of abuse with their family members/carers being willing to report this (Cooper et al., 2008). The only type of abuse associated with a higher risk of emergency department care in our study was emotional abuse, which might possibly reflect neglected or otherwise compromised co-occurring health conditions which would be more likely to result in a medical emergency. However, it is important to bear in mind that it was an isolated finding, not supported by other exposures.

In contrast to emergency care as an outcome, our results indicated consistent associations between recorded abuse and the level of subsequent face-to-face contact with mental health services. This implies a higher level of mental healthcare input required in those with recorded abuse and therefore potentially more severe presenting mental health conditions and negative health impacts (Pappadis et al., 2024). The case cohort study conducted by Rosen, Zhang et al. found that those suffering with elder abuse had approximately twice the probabilities of being admitted to hospital for treatment (Rosen et al., 2023); however, not only does abuse associate with detriment to an individual’s physical health but it is also a risk for poor mental health which may accumulate over many years (Fadeeva et al., 2024). Given that the exposure was derived from records over a 3-month period, it is possible that a higher severity index mental health condition (and/or non-recovery within that period) might have resulted in closer enquiry into potential risk factors, including abuse experiences. This would require further investigation with different study designs.

As well as higher condition severity, another potential explanation for associations with higher levels of mental healthcare contact is that individuals using mental health services in person felt more comfortable and listened to and thus were more likely to disclose this risk factor. A more personalised approach would allow them to have their queries to be answered straight away (Aslan et al., 2024) and, due to the nature of abuse being difficult to discuss for some individuals (Penhale, 2008), having the platform for face-to-face contact in a specialist service increases opportunities for professionals from a range of fields (social workers, victim support, and the police force) to assist in helping individuals who require support for abuse-related problems.

Strengths of the study lie in the large and generalisable naturalistic data afforded by the CRIS data platform as well as the novel application of NLP to ascertain recorded abuse as an exposure, hitherto impossible to investigate beyond less generalisable recruited cohort studies. Clearly, the ascertainment of abuse from the text of health records is not novel; however, the traditional approach would have required manual inspection and coding which is only feasible in relatively small samples, whereas the NLP approach used here enables this processing and ascertainment to be carried out at scale. Thus, it can be readily deployed in specific populations, such as the older adult sample investigated here, and the precision of estimates (and scope for regression analyses including multiple confounders) is increased to the extent that manifold increases in sample size outweigh any loss of precision in automated vs. manual coding. Finally, the NLP approach permits the application of future routine surveillance at scale, which would not be feasible for manual coding. Additional strengths are derived from the cost-free, catchment-level service provision in English mental healthcare (so that SLaM samples here represent nearly all mental healthcare provision to its source population), and the linkage to Hospital Episode Statistics which accesses all NHS hospitalisations in England and thus represents near-complete outcome ascertainment.

Alongside the strengths of the study, there are also some limitations to consider. As this was an observational study, the associations recognised cannot be understood as causal. In addition, patients with persistent or more severe mental health problems may have had more contact with services creating more opportunities for abuse to be recorded and acknowledged, which may present potential ascertainment bias.

The most important consideration in interpreting findings is that the NLP algorithm can only ascertain recorded abuse—hence will have missed any experiences which were not ascertained or recorded in routine clinical practice. In addition, it is important to bear in mind that the current NLP algorithm does not attempt to distinguish recent from past experiences of abuse, and hence, the exposure conflates these. However, inaccuracies in the measurement of the exposure, either arising from the NLP algorithm or the source recording of the abuse, will have resulted in obscured rather than exaggerated associations with outcomes—thus do not easily account for the strong associations with one outcome and mostly absent associations with the other. The use of NLP to classify forms of abuse could affect our findings in several ways. Training-related bias may favour forms of abuse that are more noticeable, such as physical abuse, resulting in more frequent detection (Clarysse et al., 2018).

Additional limitations of the study include the restriction of the cohort to a single South London catchment, and future studies would clearly benefit from a broader geography. In addition, as stated earlier, there is clearly a need for better understanding of pathways underlying the associations of interest, as well as other outcomes.

Finally, large numbers of subsequent mental health service contacts should not inevitably be interpreted as an indication of poorer mental health. Instead, higher contact rates may reflect greater engagement with services, safeguarding activity, or more intensive clinical support.

To conclude, older adult abuse is an important issue affecting both an individual’s mental and physical health (Alias et al., 2023). We found that this was relatively commonly recorded in routine mental healthcare and was associated with higher levels of subsequent mental healthcare use. Clinically, our findings indicate that older adults with abuse recorded represent a selection with greater subsequent mental health service needs. Although this study cannot determine that abuse caused a decline in mental health, identification of abuse should prompt consideration of broader psychological and social needs. The application of NLP to ascertain recorded abuse opens up opportunities for monitoring its recording and better understanding healthcare needs and experiences.

Further studies highlighting and raising awareness of this issue are critical so that more vulnerable groups are captured, and prevention is prioritised.

## Data Availability

The individual-level data are not publicly available because of patient confidentiality and data-governance restrictions. Access to CRIS data is available to authorised researchers subject to CRIS governance requirements and project approval. Enquiries should be directed to the CRIS administrator at cris.administrator@slam.nhs.uk.
